# Predictors of wealth-related inequality in institutional delivery: a decomposition analysis using Nepal multiple Indicator cluster survey (MICS) 2019

**DOI:** 10.1186/s12889-021-12287-2

**Published:** 2021-12-10

**Authors:** Umesh Prasad Bhusal

**Affiliations:** 1 Public Health and Social Protection Professional, Kathmandu, Nepal; 2 Melbourne School of Population and Global Health, The University of Melbourne, Melbourne, Victoria Australia

**Keywords:** Institutional delivery, Inequality, Concentration curve, Concentration index, Decomposition, Nepal, MICS

## Abstract

**Background:**

Inequality in maternal healthcare use is a major concern for low-and middle-income countries (LMICs). Maternal health indicators at the national level have markedly improved in the last couple of decades in Nepal. However, the progress is not uniform across different population sub-groups. This study aims to identify the determinants of institutional delivery, measure wealth-related inequality, and examine the key components that explain the inequality.

**Methods:**

Most recent nationally representative Multiple Indicator Cluster Survey (MICS) 2019 was used to extract data about married women (15-49 years) with a live birth within two years preceding the survey. Logistic regression models were employed to assess the association of independent variables with the institutional delivery. The concentration curve (CC) and concentration index (CIX) were used to analyze the inequality in institutional delivery. Wealth index scores were used as a socio-economic variable to rank households. Decomposition was performed to identify the determinants that explain socio-economic inequality.

**Results:**

The socio-economic status of households to which women belong was a significant predictor of institutional delivery, along with age, parity, four or more ANC visits, education status of women, area of residence, sex of household head, religious belief, and province. The concentration curve was below the line of equality and the relative concentration index (CIX) was 0.097 (*p* < 0.001), meaning the institutional delivery was disproportionately higher among women from wealthy groups. The decomposition analysis showed the following variables as the most significant contributor to the inequality: wealth status of women (53.20%), education of women (17.02%), residence (8.64%) and ANC visit (6.84%).

**Conclusions:**

To reduce the existing socio-economic inequality in institutional delivery, health policies and strategies should focus more on poorest and poor quintiles of the population. The strategies should also focus on raising the education level of women especially from the rural and relatively backward province (Province 2). Increasing antenatal care (ANC) coverage through outreach campaigns is likely to increase facility-based delivery and decrease inequality. Monitoring of healthcare indicators at different sub-population levels (for example wealth, residence, province) is key to ensure equitable improvement in health status and achieve universal health coverage (UHC).

**Supplementary Information:**

The online version contains supplementary material available at 10.1186/s12889-021-12287-2.

## Background

Maternal health is a priority public health issue, well reflected in global development agendas. Reducing preventable maternal deaths was one of the key targets of millennium development goals (MDGs). It continues to be a target of Sustainable Development Goals (SDGs). As per a global estimation done in 2017, maternal mortality ratio (MMR) was 211 per 100,000 live births [1]. By 2030, the target of SDGs is to reduce it to less than 70 per 100,000 live births [2]. Despite about a 38% reduction in global MMR since 2000, high maternal deaths is still a concern, particularly in low-and middle-income countries (LMICs), signalling the inequality in progress towards maternal health [1]. MMR is 40 times higher in the low-income countries compared to that of Europe and 60 times higher than that of Australia and New Zealand [1]. South Asia accounts for nearly one in every five global maternal deaths [1]. Low access to and utilization of health services during pregnancy and childbirth such as antenatal care (ANC), institutional delivery and skilled birth attendants (SBAs) are key factors responsible for a higher number of maternal deaths in this region [3]. These deaths, to a larger extent, results from complications during delivery such as haemorrhage, sepsis, unsafe abortion, obstructed labour, and hypertensive disorders that could be prevented by switching from home to institutional delivery [1, 3–6]. The skilled attendance at delivery in a hygienic environment and timely access to emergency care abate the risk of mortality or serious complications for both mother and newborn [7].

Equity in access to and utilization of healthcare services has received increased attention lately. It is one of the health system goals highlighted in the health system framework proposed by World Health Organization (WHO) in 2007 [8, 9], and a crucial element of universal health coverage (UHC) embodied in SDGs [2, 10]. However, inequality in the distribution of access to and utilization of maternal health services both between and within countries continues to be a major concern for LMICs [7, 11, 12]. Studies from different countries have shown that maternal health service utilization is disproportionately higher among women from wealthier households compared to their poorer counterparts; those living in an urban area compared to rural counterparts; those with higher education compared to non-educated counterparts; those belonging to the accessible geographical areas compared to remote counterparts [3, 6, 7, 13–18]. Different socio-economic and demographic factors interact with each other and aggravate the situation of inequality.

The maternal health indicators at the national level have markedly improved in the last couple of decades in Nepal. The percent of women aged 15-49 years delivered in health institutions has increased from 8 in 1996 to 57 in 2016 [19]. Similarly, percent of women receiving more than four antenatal care (ANC) has increased from 14 in 2001 to 69 in 2016 [19]. The maternal mortality ratio (MMR) has decreased from 539 maternal deaths per 100,000 live births to 239 between 1996 and 2016 [19]. This progress is attributed to a mix of supply and demand-side financing strategies introduced by Government of Nepal (GoN) since the 1990s [20–24]. Establishment of birthing centres (BCs) and basic/comprehensive emergency obstetric centres (BEOCs/CEOCs) in remote and rural areas; skilled birth attendant training to nursing staffs and doctors of BCs and EOCs; expansion of blood transfusion services; strengthening of the referral services are key examples of supply-side financing. Likewise, maternity incentive scheme introduced in 2005 (transport incentive to mothers who deliver in health facilities) and revised subsequently in 2006 (user fees removed in all facilities in 25 districts with low human development index), 2009 (nationwide user fee removed and renamed as *Aama* program), and 2012 (incentive for completing recommended four ANC visits followed by institutional delivery introduced in 2009 merged with *Aama* program) are examples of demand-side financing.

However, the progress in maternal health in Nepal is not uniform across population from different geography and socio-economy. The evidence shows that the investment made in maternal health disproportionately favours women belonging to: an urban area, educated group, wealthy households, privileged ethnic group [25]. Despite the focus of GoN towards enhancing equitable distribution and utilization of health services through policies and sector strategies, progress in narrowing the socio-economic inequality was not uniform across seven provinces in Nepal as demonstrated by the further analyses of nationally representative household surveys conducted before 2016 [4, 20, 24, 26].

GoN is committed to UHC and SDGs to be achieved by 2030. Hence, measuring health service utilization from the equity perspective using the most recent survey data is essential to ensure fair progress across population sub-groups. It is also of paramount importance to analyze the determinants of inequality so that evidence-based policy intervention could be taken by the policymakers. There is a dearth of studies that examine the determinants of inequality in maternal health in Nepal. The objective of this paper is three-fold: (i) to analyze the determinants of institutional delivery in Nepal using the most recent nationally representative household survey; (ii) to measure the socio-economic inequality in the use of institutional delivery services; (iii) to identify the main components that explain socio-economic inequality in institutional delivery through decomposition analysis.

## Methods

### Study design and setting

This study analyzed the Multiple Indicator Cluster Survey (MICS) 2019 data set. The survey was conducted in Nepal by the Central Bureau of Statistics (CBS) in technical and financial support from UNICEF. MICS is a nationally representative cross-sectional survey that aims to monitor the situation of women and children by capturing the information on health, education, social protection, environment, domestic violence along with the socio-economic, demographic and geographic characteristics at the individual and household level. The sampling frame of the Nepal MICS 2019 was based on the National Population and Housing Census 2011. The frame consisted of a complete list of all census wards created in 2011 and updated in 2018 to account for the current administrative structure of Nepal. The survey employed a multistage, stratified cluster probability sampling design to establish a representative sample of households at the national and province level. Within each province, the urban and rural areas were defined as the main sampling strata. The sample of households was selected in the following stages: (i) within each stratum, a specified number of census enumeration areas (EAs) or clusters were selected systematically with probability proportional to size (then listing of the household was done for the selected EAs) (ii) the sample of households was selected from the sampled EAs. In total, 25 households were selected from each sampled EA through a systematic random sampling method. For this round of survey, a total of 512 EAs and 12,800 households were selected. Out of which; 12,655 households, 14,805 women (15-49 years), and 5501 men (15-49 years) were successfully interviewed. Details of the MICS design and methodology are described elsewhere [27].

### Study population

**Fig. 1:**
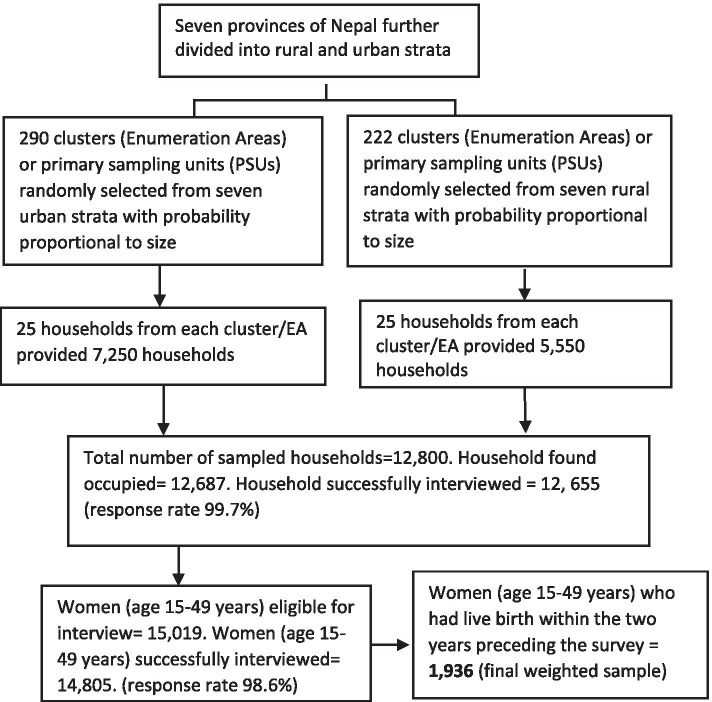
Flow chart showing selection of study population (Nepal MICS 2019)

This study used data from married women (15-49 years) who had a live birth within the two years preceding the survey. In case of multiple births by the selected women within the two years, the data analysis was conducted for their most recent live birth. A total of 1936 women (unweighted count = 2500) were eligible to be included in this study. Selection of the study population is summarized in a flow chart (Fig. 1).

### Study variables

The outcome variable used in this study was institutional delivery. It was categorized as “1” if women delivered their baby at health facility (both public or private) and “0” if women delivered their baby at home. The independent or explanatory variables included in this study are: age of women at the time of survey, number of births (parity), education status of women, exposure to media, four or more ANC visits for the most recent live birth, education status of household head, sex of household head, religion, ethnicity, area (rural and urban), province and wealth index quintile.

Age of women was categorized into four groups (1: 15-19 years; 2: 20-29 years; 3: 30-39 years; 4: 40-49 years). Religion was broadly categorized as Hindu and non-Hindu. More than 100 castes recorded during the survey were re-classified into four caste/ethnic groups based on Population Monograph of Nepal 2014 [28] (1: Brahmin, Chhetri and Madhesi; 2: Janajati from mountain, hill, and *terai* (plain southeast belt) and Newer; 3: Dalits from mountain hill and *terai* and Muslims; 4: rest (Marwadi, Bangali etc) as others. Education status of women and household head was categorized into four groups (0: without formal education; 1: primary education (grade one to five); 2: secondary education (grade six to ten); 3: higher secondary education and above (grade 11, 12 and above). Exposure to media was classified based on whether women were exposed to at least one source of mass media (0: no exposure; 1: limited exposure where women either listen to radio or watch TV or read magazine/newspaper less than once in a week; 2: exposure where women either listen to radio or watch TV or read magazine/newspaper at least once in a week to almost every day). Wealth index is a composite indicator of wealth and is commonly used as a proxy measure of socio-economic status of a household. We used wealth index available in Nepal MICS 2019 dataset. To construct the wealth index, principal components analysis was performed by using information on the ownership of consumer goods, dwelling characteristics, water and sanitation, and other assets and durables that are related to the household’s wealth [27]. Since the wealth index provides an ordinal interpretation, it was used as a ranking variable of households.

### Method of analysis

#### Descriptive and regression analyses

The characteristics of the women included in this study were described by using frequency and percentage. Bivariate and multivariate logistic regression models were used to assess the association of independent variables with institutional delivery. STATA 16 for the statistical analysis was used. Complex survey design was declared by using *svyset* command to account for sampling weights, clustering and stratification in the sampling design. Variance Inflation Factors (VIFs) were used to examine multicollinearity among covariates before building regression models. All covariates had VIFs less than 3.5 (maximum: 3.37; minimum: 1.06; average: 1.82). The details of the VIFs are presented in Supplementary Table 1. Test for specification error [29] was done to confirm the assumption that the logit of the outcome variable is a linear combination of the independent variables. This involved two steps: (i) whether logit is the correct function to use in this regression model; (ii) whether all the relevant (but no extraneous) variables are included. The Stata command *linktest* was employed to test for specification error. The output of the command *linktest* is provided in Supplementary Table 2. Goodness of fit test accounting for survey design [30] was used to assess the fitness of multivariate logistic regression model. There was no evidence of lack of fitness (F-adjusted test statistic: 1.5; *p*-value: 0.144). All analyses were weighted.

#### Inequality measurement

The concentration curve (CC) and concentration index (CIX) in their relative formulation (with no correction), were used to analyze the inequality in the use of health services (institutional delivery) across socio-economic characteristics of the population (women) [31]. The CIX presented in this paper corresponds to the horizontal inequity since every woman in the study were assumed to have an equal need for institutional delivery. While producing CC, cumulative proportion of women ranked by wealth index score (poorest first) was plotted on the x-axis against the cumulative proportion of institutional delivery on the y-axis. The 45-degree inclination from the origin showed perfect equality. If the CC overlaps with the line of equality, use of institutional delivery is equal among women. However, if the CC subtends the line of equality below (above), then inequality in the use of institutional delivery exists and is biased towards women belonging to low (high) socio-economic status. The further the CC subtends from the line of equality, the greater the degree of inequality.

To quantify the magnitude of wealth-related inequality, CIX was calculated. CIX is defined as twice the area between the line of equality and CC [31]. The following are the advantages of using CIX as a measure of inequality index in healthcare: it takes socio-economic dimension of healthcare inequalities into account since the classification of individuals is according to the socio-economic status, instead of their health status; it captures the experience of the whole population and; it is sensitive towards the changes in population distribution across socio-economic groups [15]. The CIX takes a value between − 1 and + 1. When the institutional delivery is equally distributed across socio-economic groups, CIX takes the value of 0. A positive value of CIX implies that the use of institutional delivery is concentrated among the higher socio-economic groups (pro-rich). Conversely, a negative value of CIX suggests that the use of institutional delivery is concentrated among the lower socio-economic groups (pro-poor).The calculation of CIX was done by using “convenient covariance” formula described by O’Donnell et al. [31], as shown in eq. 1 below.

1 $$CIX=\frac{2}{\mu}\mathit{\operatorname{cov}}\left(h,r\right)$$

Here *h* is the health sector variable, *μ* is its mean, and *r* = *i*/*N* is the fractional rank of individual *i* in the living standards distribution, with *i* = 1 for the poorest and *i* = *N* for the richest. The user-written STATA commands *lorenz* [32] and *conindex* [33] were used to produce CC and measure CIX, respectively.

#### Decomposition of CIX

The decomposition of the relative CIX was performed to calculate the portion of inequality that is due to the inequality in underlying determinants. The technique explained by Wagstaff et al. [34] and O’Donnell et al. [31] were followed for the analysis and interpretation of results. The contribution of individual determinant of institutional delivery to the overall wealth-related inequality was calculated as the product of sensitivity of institutional delivery with respect to the determinant (elasticity) and the degree of wealth-related inequality in that determinant (CIX of determinant). Part of the CIX not explained by the determinants was presented as residual. ADePT (version 6) software platform for automated economic analysis developed by World Bank was used for required calculations. Since the institutional delivery is a binary outcome variable, the non-linear model was specified while conducting the analysis. Selection of variables for the decomposition of CIX was based on the results of multivariate logistic regression (statistical significance), policy relevance and literature review of empirical studies [15, 17, 35].

## Results

### Descriptive summary

Table 1 shows the descriptive statistics for socio-economic and demographic characteristics of women aged 15-49 years disaggregated by the place of delivery (home delivery versus institutional delivery). Overall, out of 1936 women, about 78.2% delivered in health institution and 21.8% delivered in home. Most of the women in this study belonged to age group 20-29 years, had one child, did four or more ANC visits, had secondary education, were exposed to mass media, belonged to urban residence, had male as a household head, were from upper ethnic group, and were Hindu. Similarly, most of the women had household head without formal education, belonged to Province 2, and were from the poorest wealth quintile.

**Table 1:** Socio-economic and demographic characteristics of women by place of delivery (*N* = 1936)

Variables	Frequency (weighted) (%)		Home delivery (%) (***n*** = 422)	Institutional delivery (%) (***n*** = 1514)	p-value for Chi-square test
**Age of women (years)**					
15-19	200 (10.3)		9.1	10.7	0.097
20-29	1307 (67.5)		64.7	68.3	
30-39	386 (19.9)		22.9	19.1	
40-49	43 (2.2)		3.3	1.9	
**Parity**					
One	843 (43.5)		21.4	49.7	< 0.001
Two	640 (33.0)		30.9	33.6	
Three	250 (12.9)		25.5	9.4	
Four or more	204 (10.5)		22.2	7.3	
ANC visit*					
Less than four	342 (18.5)		40.9	13.3	< 0.001
Four or more	1507 (81.5)		59.1	86.7	
**Education status of women**					
No formal education	404 (20.9)		43.7	14.5	< 0.001
Primary education (Grade1-5)	260 (13.4)		17.4	12.3	
Secondary education (Grade 6-10)	813 (42.0)		36.4	43.6	
HSS and above (Grade 11/12 and above)	460 (23.7)		2.5	29.7	
**Exposure to mass media**					
No exposure	550 (28.4)		49.8	22.4	< 0.001
Limited exposure	186 (9.6)		9.5	9.7	
Exposure	1199 (61.9)		40.6	67.9	
**Area of residence**					
Rural	667 (34.5)		52.6	29.4	< 0.001
Urban	1269(65.5)		47.4	70.6	
**Sex of the household head**					
Female	418 (21.6)		16.5	23.0	0.005
Male	1518 (78.4)		83.5	77.0	
**Ethnicity**					
Brahmin, Chhetri and Madhesi	822 (42.4)		41.0	42.9	< 0.001
Janajati and Newar (Mountain, Hill and Terai)	674 (34.8)		29.9	36.2	
Dalit (Mountain, Hill and Terai) and Muslim	405 (20.9)		28.6	18.8	
Others (eg. Marwadi, Bangali)	35 (1.8)		0.6	2.1	
**Religion**					
non-Hindu	309 (16.0)		21.1	14.6	0.005
Hindu	1627 (84.0)		78.9	85.4	
**Education status of household head**					
No formal education	769 (39.7)		51.6	36.4	< 0.001
Primary education (Grade 1-5)	391 (20.2)		22.5	19.6	
Secondary education (Grade 6-10)	568 (29.3)		23.5	31.0	
HSS or above (Grade 11/12 and above)	208 (10.7)		2.4	13.0	
**Province**					
Province 1	304 (15.7)		15.0	15.9	< 0.001
Province 2	414 (21.4)		34.9	17.6	
Bagmati Province	382 (19.7)		9.7	22.5	
Gandaki Province	150 (7.8)		3.1	9.1	
Lumbini Province	369 (19.1)		18.9	19.1	
Karnali Province	131 (6.8)		11.5	5.4	
Sudurpaschim Province	186 (9.6)		6.8	10.4	
**Wealth index quintile**					
Poorest	438 (22.6)		43.9	16.7	< 0.001
Poor	408 (21.1)		25.2	19.9	
Middle	384 (19.8)		17.5	20.4	
Richer	383 (19.8)		11.0	22.2	
Richest	324 (16.7)		2.4	20.7	
**Total**	**1936**		**21.8**	**78.2**	

Abbreviation: HSS = higher secondary education

*doesnot sum to 1936 due to missing values

**Fig. 2:**
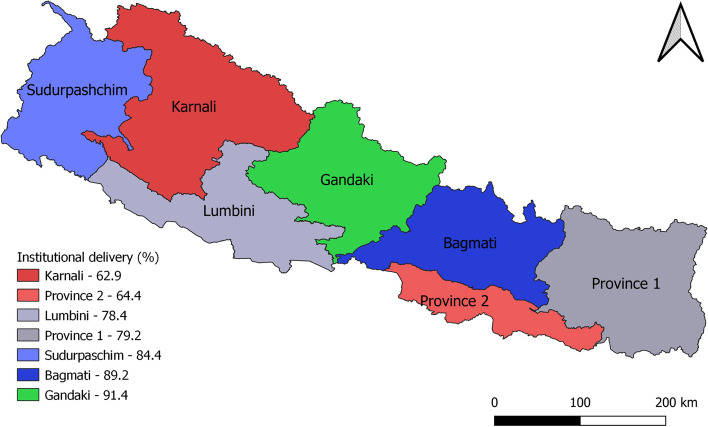
Map of Nepal showing Province wise percentage of institutional delivery. Map was created using QGIS 3.16. Shapefile was accessed from publicly available source

Proportion of home delivery and institutional delivery was identical among different age groups. Women with one or two births, four or more ANC visits, formal education, exposed to mass media, from urban area were more likely to deliver in health institution. Higher proportion of women belonging to upper caste and Hindu religion had institutional delivery compared to Dalit and non-Hindu women. The education status of household head was negatively associated with the home delivery. Institutional delivery was concentrated more in Bagmati province, followed by Lumbini Province, Province 2, and Province 1. In contrast, home delivery was concentrated in Province 2, followed by Lumbini Province and Province 1. Likewise, institutional delivery was distributed more in richer quintiles in comparison to poorer counterparts. Except for age of women, there was strong evidence of an association between socio-economic and demographic characteristics of women and place of delivery (demonstrated by *p*-value for chi-square test, Table 1). The map of Nepal showing province was status of institutional delivery as a percentage of total delivery in that province is shown in Fig. 2.

### Results from the regression model

Table 2 presents the estimate and the corresponding 95% confidence interval (CI) for the bivariate and multivariate regression models as unadjusted odds ratio (OR) and adjusted OR, respectively. Both the bivariate and multivariate analyses showed that women who have completed four or more ANC visits; attained higher education; from urban residence; belonging to Hindu religion; and from wealthier quintiles were more likely to deliver in health institution in comparison to their respective counterparts. Conversely, the lower odds of institutional delivery were found for multiparous women; those living in male-headed household; and women belonging to Province 2 in comparison to the respective reference groups. Some variables (exposure to mass media, ethnicity, education status of household head, belonging to Bagmati, Gandaki or Karnali Province) that showed statistically significant association with institutional delivery in bivariate analyses did not show such association in multivariate analysis. Likewise, few associations were apparent only in the multivariate models (age of women, belonging to Sudurpaschim Province).

**Table 2:** Determinants of institutional delivery in Nepal (MICS 2019), *N* = 1936

Variables	Odds ratio (95% Confidence Interval)	p-value		***p***-value
	Unadjusted OR		Adjusted OR	
**Age of women (years)**				
15-19	1.00		1.00	
20-29	0.89 (0.60-1.33)	0.578	1.58 (0.98-2.55)	0.059
30-39	0.71 (0.46-1.09)	0.116	2.39 (1.29-4.42)	0.006
40-49	0.50 (0.25-1.01)	0.054	2.66 (1.04-6.78)	0.041
**Parity**				
One	1.00		1.00	
Two	0.47 (0.35-0.63)	< 0.001	0.41 (0.28-0.59)	< 0.001
Three	0.16 (0.11-0.22)	< 0.001	0.21 (0.14-0.32)	< 0.001
Four or more	0.14 (0.10-0.21)	< 0.001	0.27 (0.15-0.49)	< 0.001
**ANC visit**				
Less than four	1.00		1.00	
Four or more	4.51 (3.39-6.01)	< 0.001	2.54 (1.84-3.50)	< 0.001
**Education status of women**				
No formal education	1.00		1.00	
Primary education (Grade 1-5)	2.13 (1.54-2.95)	< 0.001	1.10 (0.68-1.77)	0.698
Secondary education Grade 6-10)	3.61 (2.68-4.86)	< 0.001	1.17 (0.75-1.80)	0.489
HSS and above (Grade 11/12 or above)	36.11 (20.01-65.18)	< 0.001	5.18 (2.39-11.23)	< 0.001
**Exposure to mass media**				
No exposure	1.00		1.00	
Limited exposure	2.25 (1.52-3.34)	< 0.001	1.19 (0.74-1.90)	0.471
Exposure	3.71 (2.86-4.82)	< 0.001	1.01 (0.71-1.45)	0.944
**Area of residence**				
Rural	1.00		1.00	
Urban	2.67 (1.99-3.58)	< 0.001	1.80 (1.29-2.52)	0.001
**Sex of the household head**				
Female	1.00		1.00	
Male	0.66 (0.49-0.88)	0.005	0.68 (0.47-0.98)	0.041
**Ethnicity**				
Brahmin, Chhetri and Madhesi	1.00		1.00	
Janajati and Newar (Mountain, Hill and Terai)	1.16 (0.84-1.60)	0.370	1.14 (0.77-1.68)	0.508
Dalit (Mountain, Hill and Terai) and Muslim	0.63 (0.46-0.86)	0.004	1.25 (0.86-1.80)	0.236
Others (eg. Marwadi, Bangali)	3.42 (1.13-10.37)	0.030	2.32 (0.72-7.44)	0.157
**Religion**				
non-Hindu	1.00		1.00	
Hindu	1.57 (1.15-2.15)	0.005	1.69 (1.16-2.46)	0.006
**Education status of household head**				
No formal education	1.00		1.00	
Primary education (Gr. 1-5)	1.23 (0.92-1.64)	0.154	0.87 (0.59-1.28)	0.481
Secondary education Gr. 6-10)	1.87 (1.42-2.46)	< 0.001	0.98 (0.71-1.35)	0.891
HSS and above (Gr. 11/12 and above)	7.58 (4.11-13.96)	< 0.001	1.89 (0.93-3.82)	0.076
**Province**				
Province 1	1.00		1.00	
Province 2	0.48 (0.29-0.79)	0.004	0.42 (0.25-0.72)	0.002
Bagmati Province	2.18 (1.22-3.92)	0.009	1.29 (0.69-2.41)	0.431
Gandaki Province	2.79 (1.43-5.44)	0.003	1.35 (0.64-2.83)	0.428
Lumbini Province	0.96 (0.57-1.59)	0.861	0.80 (0.45-1.42)	0.449
Karnali Province	0.45 (0.26-0.76)	0.003	1.05 (0.53-2.06)	0.894
Sudurpaschim Province	1.43 (0.78-2.60)	0.244	2.13 (1.06-4.29)	0.033
**Wealth index quintile**				
Poorest	1.00		1.00	
Second	2.07 (1.52-2.83)	< 0.001	2.63 (1.68-4.12)	< 0.001
Middle	3.07 (2.09-4.50)	< 0.001	4.62 (2.73-7.82)	< 0.001
Richer	5.33 (3.49-8.13)	< 0.001	5.39 (2.99-9.69)	< 0.001
Richest	22.55 (10.45-48.64)	< 0.001	7.19 (2.80-18.46)	< 0.001

Abbreviation: MICS = Multiple Indicator Cluster Survey; OR = odds ratio; HSS = higher secondary education

Women in age group 30 to 39 years were about two times (adjusted OR = 2.39; 95% CI: 1.29-4.42) more likely to deliver in health institution compared to those in age group 15 to 19 years. Similarly, women in age group 40 to 49 years were about two and a half times (adjusted OR = 2.66; 95% CI: 1.04-6.78) more likely to deliver in health institution compared to those in age group 15 to 19 years. Women who had delivered two, three, and four or more children already were significantly less likely to deliver in health institution compared to those who had only one child ever born. Women who had received four or more antenatal care (ANC) visits were nearly two and a half times more likely to deliver in health institution (adjusted OR = 2.54; 95% CI: 1.84-3.50) compared to those with fewer ANC visits. Women with higher secondary education or above were nearly five times more likely to deliver in health institution (adjusted OR = 5.18; 95% CI: 2.39-11.23) compared to those without formal education. Similarly, women residing in urban area had greater odds of delivering in health institution (adjusted OR = 1.80; 95% CI: 1.29-2.52) compared to those living in rural setting. However, women from male-headed households were less likely to deliver in health facility (adjusted OR = 0.68; 95% CI: 0.47-0.98) compared to female-headed households. Regarding religion, Hindu women were more likely to deliver in health institution (adjusted OR = 1.69; 95% CI: 1.16-2.46) compared to non-Hindu women. Similarly, women from Sudurpaschim Province were more likely to deliver in health institution (adjusted OR = 2.13; 95% CI: 1.06-4.29) compared to those from Province 1. However, women from Province 2 were less likely to deliver in health institution.

Women belonging to higher wealth index quintile had greater odds of delivering in health institution compared to poorest quintile. Women from second, middle, richer and richest wealth index quintile were more than two times, four times, fifth times and seventh times more likely to deliver in health institution compared to poorest wealth quintile (reference), respectively.

### Results from the measures of inequality

**Fig. 3:**
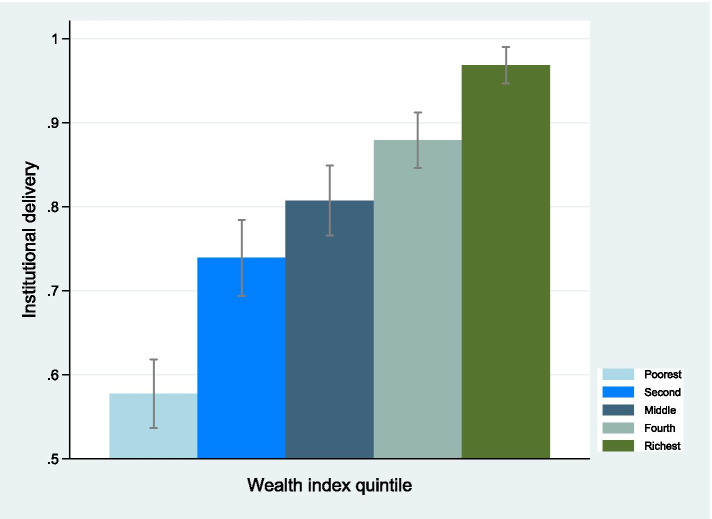
Institutional delivery over wealth index quintiles

**Fig. 4:**
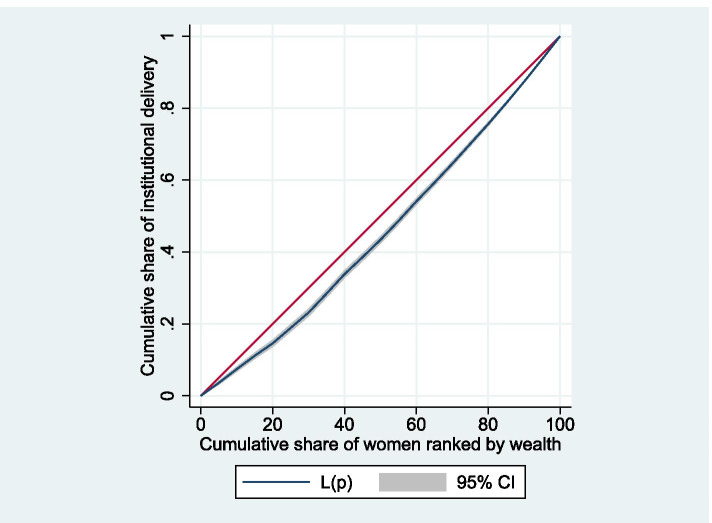
Concentration curve for institutional delivery against wealth rank

Figure 3 depicts average institutional delivery (with 95% confidence interval) over wealth index quintiles with respect to the total delivery in that quintile. Just under 60% of women in the poorest wealth quintile delivered in health institutions compared to about 95% in the richest counterpart. The graph demonstrates that institutional delivery increases monotonically in moving from women in the poorest wealth quintile to the richest wealth quintile. Figure 4 shows the inequality in institutional delivery by wealth status. Since the concentration curve is below the line of equality, the institutional delivery was disproportionately higher among women from wealthy groups. A positive estimated relative CIX of 0.097 (standard error: 0.008; *p* < 0.001) indicates that the institutional delivery was concentrated among the wealthier women in comparison to their poor counterparts

### Decomposition of the relative concentration index (CIX)

Table 3 presents the result of decomposition analysis of the relative CIX used to ascertain the contributions of different determinants towards wealth-related inequality in institutional delivery. The major contribution to the inequality was from the wealth status of household women belongs (53.20%), followed by education (17.02%), area of residence (8.64%), and ANC visit (6.84%) The residual contribution (13.70%) represents the amount of wealth-related inequality not explained by the determinants used in the analysis.

**Table 3:** Decomposition of concentration index (CIX)

Variables	Elasticity	CIX of determinant	Contribution to total CIX	Percent contribution
Age	0.0537	0.0113	0.0006	0.62
ANC visit	0.1730	0.0383	0.007	6.84
Education (women)	0.1467	0.1125	0.017	17.02
Residence	0.1246	0.0672	0.008	8.64
Wealth	0.1896	0.2722	0.052	53.20
Residual			0.0133	13.70
**Total**			**0.097**	

Abbreviation: CIX = concentration index

## Discussion

This study analyzed the determinants of institutional delivery in Nepal using the most recent MICS 2019. The odds of Nepalese women giving birth in health institutions with respect to their socio-economic and demographic characteristics were measured. Further, the wealth-related inequality in institutional delivery was calculated along with a decomposition analysis to find out the key determinants that explain the inequality [31]. The study found that age of women, parity, four or more ANC visit, education status of women, area of residence, sex of household head, religious belief, province, and wealth index quintile were significant determinants for the institutional delivery. The institutional delivery was disproportionately higher among women belonging to wealthy households. The decomposition of the concentration index showed that the wealth-related inequality was explained mostly by household wealth, education status of women, urban residence, and ANC visits.

The odds of institutional delivery increased with the increase in age of women. The women above age 30 years were more than two times more likely to have institutional delivery compared to that of age below 15-19 years. This finding corroborates previous study that analyzed first-order births in 34 countries of sub-Saharan Africa and found that older age at birth was associated with significantly higher odds of facility-based delivery [36]. Finding from this study aligns well also with the study from Bangladesh [37]. However, the non-significant association was obtained in few studies from Nepal, Pakistan and Ethiopia [3, 38, 39] and even negative association was obtained in a previous study from Nepal [40]. Further studies are needed to investigate the association between age of women and institutional delivery. The likelihood of institutional delivery decreased with an increase in parity. This result support finding from similar studies conducted in developing countries that have shown that experienced mothers were less likely to opt for facility-based delivery [3, 7, 39, 41]. One possible explanation for the low uptake of institutional delivery among high parity women is that women with birth history may develop confidence from the knowledge and experience acquired from earlier pregnancies and therefore are less motivated to opt for services from health facilities [7]. The odds of institutional delivery was twice in women who had completed four or more ANC visits. Positive association between the antenatal visits and facility-based delivery was found in previous studies conducted in Kenya, Ethiopia, Nepal, Bangladesh, and Pakistan [3, 4, 6, 37, 39–42]. The counselling on birth preparedness received from health workers during the antenatal visits could be one of the reasons.

The women with higher education were significantly more likely to deliver in health institution compared to women without formal education. Similar findings were obtained from studies conducted in other developing countries, establishing education as a significant determinant of facility-based delivery [3, 4, 14, 15, 38, 40, 41, 43]. This association could be attributed to the fact that educated women are more likely to have a better understanding of risks associated with childbirth and benefits of using skilled healthcare compared to uneducated women. This evidence implies that inequality in institutional delivery could be reduced with appropriate intervention targeted to educate women. To reduce the barriers in uptake of maternal health services, GoN has been implementing Birth Preparedness Package (BPP) through health workers and Female Community Health Volunteers (FCHVs) in the community since the early 2000s as part of the safe motherhood program [44]. BPP educates pregnant women, their families, and communities to plan for normal pregnancy, delivery, and postnatal period and creates demand for healthcare through inter-personal communication using specially designed cards and flipcharts. So, more focused outreach education and awareness campaigns are key to further reduce the inequality in use of maternal healthcare services between educated and uneducated mothers. Study conducted by Karkee et al. using nationally representative dataset from 2011,however, found no significant association between education of women and institutional delivery [39].

The women from urban area were nearly two times more likely to opt for institutional delivery compared to the ones from rural counterparts. The findings from this study corroborate those of prior studies, where it was shown that urban residence of mother was associated with an increase in the use of institutional delivery [3, 15, 17, 38, 39, 41, 45]. In general, women from urban area have better access to healthcare system due to better transport, well-equipped hospitals and less distance between residence and health facility [15, 17]. Studies based in rural settings of Nepal have identified access to birthing facilities, perception regarding the quality of healthcare, lack of transportation, poor infrastructure and equipment at birthing centres as key barriers to access facility-based delivery services [46–48].

Women from Province 2 were significantly less likely to deliver in health institution compared to women from Province 1. Similar finding was obtained from a study using nationally representative dataset of 2016 [40]. Province 2 is *terai* (plain southeast belt) of Nepal and generally falls behind other provinces in terms of public health coverage indicators [20] and health infrastructure. As per the evidence from nationally representative surveys it had the lowest percentage of facilities providing normal vaginal delivery [49] and lowest mean general health service readiness score [50]. It observed the lowest annual change in Human Development Index (HDI) since 1996 and had one of the third lowest HDI of 0.485 (national average = 0.522) in 2011 [51]. Focused measures that address the unique socio-economic positioning are urgent to bring maternal health indicators of Province 2 at par with the national average.

Institutional delivery increased monotonically in moving from women in poorest wealth quintile to richest wealth quintile. Analysis of concentration curve and concentration index revealed a pro-rich inequity in institutional delivery. The result from this study is consistent with findings from similar studies where it was shown that better socio-economic condition of mothers was associated with an increase in the use of maternal healthcare services [7, 14–18, 39–41, 52, 53]. Women belonging to higher socio-economic group have a better chance to visit health facility and when required, make payment for the expenses related to travel and medical care [15, 53]. Various studies indicate the potential reasons for disproportionally lower coverage of maternal health services among women from lower socio-economic group: direct and indirect cost related to healthcare including travel expenses and opportunity cost; perceived quality of care in public facilities; perceived importance of seeking formal healthcare during pregnancy and childbirth [7, 15, 16]. This indicates that the efforts of government since the 1990s to address barriers posed by Nepalese, mainly the poor households, through various supply and demand-side financing are still insufficient. However, as observed in earlier analysis conducted using four rounds of Nepal Demographic and Health Survey (NDHS: 2001, 2006, 2011, 2016), the socio-economic inequality concerning institutional delivery between the socio-economic groups measured by relative CIX has, on average, narrowed over this period [20]. The relative CIX obtained from these four rounds of NDHS were 0.56, 0.48, 0.35 and 0.19, respectively [20]. The analysis presented in this paper using the data from MICS 2019 has shown that the relative CIX for institutional delivery has further narrowed down to 0.097. So, the investment made by GoN looks working, but it should be more focused on benefiting the lower socio-economic group (in contrast to the current blanket approach with more emphasis on national targets), to further reduce the inequality gap. The decomposition analysis found that household wealth status contributes significantly towards the inequality in institutional delivery (53.2%), followed by women’s education (17.02%.), urban residence (8.64%) and ANC visit (6.84%). This implies that the future policies and strategies need to be pro-poor, pro-rural and that need to focus more on educating women and families for increased ANC uptake. The result of decomposition analysis presented in this paper is consistent with similar studies from developing countries [15, 17, 18, 54].

Few variables that did not demonstrate a significant association with institutional delivery in this study showed a statistically significant association in studies conducted in other settings. Unlike Pulok et al. [7], Ketemaw et al. [38] this study found no statistically significant association between media exposure and institutional delivery. Similarly, unlike Atake [15], Pulok et al. [7] and Obiyan and Kumar [55], this study found no statistically significant association between education status of the household head (usually male/husband in Nepalese context) and institutional delivery. More studies might be required to ascertain these associations.

### Strength of this study

This study has used the most recent nationally representative household survey conducted in 2019. Rigorous statistical methods to calculate the odds of enrollment controlling for relevant socio-economic and demographic variables were employed. Further, this study adds to the current body of literature from Nepal by providing the composite measure of inequality using standard techniques and performing decomposition analysis to identify key determinants that explain the inequality in the use of institutional delivery in Nepal.

### Limitation of this study

First, the list of independent variables included in this study may not be an exhaustive one. The variables such as employment status of women, distance to the nearest institution with the birthing facility, cost (direct or indirect) associated with institutional delivery, understanding of the importance of safe delivery could not be included in this analysis due to the unavailability of such data in this round of MICS. Second, since this study is cross-sectional, it could not establish any causal relationship between the variables under study and institutional delivery. Notwithstanding, this study has elicited empirical evidence on socio-economic inequality and its predictors regarding institutional delivery which have policy relevance to countries with similar socio-economic context to Nepal.

## Conclusion

Age of women, parity, four or more ANC visit, education status of women, area of residence, sex of household head, religious belief, province and household wealth were found to be important predictors of institutional delivery in Nepal. Institutional delivery was found to be disproportionately higher among women belonging to wealthy households. The decomposition of the concentration index showed that wealth-related inequality was explained mostly by socio-economic status of household, education status of women, residence, and ANC visit. The pro-poor strategies are urgent to further reduce the existing inequality between women belonging to different socio-economic groups. The strategies should focus on raising the education level of women especially from the rural and backward province (Province 2). Increasing antenatal care coverage through the outreach campaign is likely to increase facility-based delivery and reduce the gap between poor and wealthy women. Monitoring of healthcare indicators at different sub-population level (for example wealth, residence, education, province) is key to ensure equitable improvement in health status and achieve universal health coverage (UHC) by 2030.

## Supplementary Information

**Additional file 1: Supplementary table 1. **Variance Inflation factors (VIFs) of variables included in the multivariate logistic regression model. **Supplementary Table 2.** Results of specification error test using *linktest* command in Stata

## Data Availability

Publicly available data were used that are accessible from the MICS website (https://mics.unicef.org/surveys) upon request.

## Abbreviations

ANC: Antenatal care
BC: Birthing centre
BEOC: Basic emergency obstetric care
BPP: Birth preparedness package
CBS: Central Bureau of Statistics
CC: Concentration curve
CEOC: Comprehensive emergency obstetric care
CIX: Concentration index
DHS: Demographic and health survey
FCHV: Female community health volunteers
GoN: Government of Nepal
HDI: Human development index
LMIC: Low-and middle-income country
MDG: Millennium development goal
MICS: Multiple indicator cluster survey
MMR: Maternal mortality rate
OR: Odds ratio
SBA: Skilled birth attendant
SDG: Sustainable development goal
UHC: Universal health coverage
UNICEF: United Nations Children’s Fund
WHO: World Health Organization
