# Supplementary material for: Predictors of wealth-related inequality in institutional delivery: a decomposition analysis using Nepal multiple Indicator cluster survey (MICS) 2019
Source: BMC Public Health. 2021 Dec 10;21:2246. doi: 10.1186/s12889-021-12287-2 (PMC8665495; doi:10.1186/s12889-021-12287-2)
Supplement: Supplementary file 1 — Additional file 1: Supplementary table 1. Variance Inflation factors (VIFs) of variables included in the multivariate logistic regression model. Supplementary Table 2. Results of specification error test using linktest command in Stata [file 12889_2021_12287_MOESM1_ESM.docx]

**Supplementary Information**

**Supplementary table 1** Variance Inflation factors (VIFs) of variables included in the multivariate logistic regression model

| **Variables** | **VIFs** |
| --- | --- |
| **Age of women in years** |  |
| 20-29 years | 2.87 |
| 30-39 years | 3.37 |
| 40-49 years | 1.38 |
| **Parity** |  |
| Two | 1.4 |
| Three | 1.48 |
| Four or more | 1.76 |
| **ANC visit** (four or more) | 1.14 |
| **Education status of women** |  |
| Primary education | 1.77 |
| Secondary education | 2.93 |
| HSS and above | 3.2 |
| **Exposure to mass media** |  |
| Limited exposure | 1.29 |
| Exposure | 1.73 |
| **Area of residence** (urban) | 1.25 |
| **Sex of the household head** (male) | 1.08 |
| **Ethnicity** |  |
| Janajati and Newar (Mountain, Hill and Terai) | 1.59 |
| Dalit (Mountain, Hill and Terai) and Muslim | 1.38 |
| Others | 1.06 |
| **Religion** (Hindu) | 1.23 |
| **Education status of household head** |  |
| Primary education | 1.33 |
| Secondary education | 1.44 |
| HSS and above | 1.52 |
| **Province** |  |
| Province 2 | 2.1 |
| Bagmati Province | 2.07 |
| Gandaki Province | 1.73 |
| Lumbini Province | 1.92 |
| Karnali Province | 2.08 |
| Sudurpaschim Province | 1.94 |
| **Wealth index quintile** |  |
| Poor | 1.88 |
| Middle | 1.96 |
| Richer | 2.18 |
| Richest | 2.4 |
| **Mean VIF** | **1.82** |

**Supplementary table 2** Results of specification error test using *linktest* command in Stata

| **Institutional delivery** | **Coefficient** | **95% confidence interval** | ***p*-value** |
| --- | --- | --- | --- |
| **_hat** | 0.968 | 0.779-1.157 | <0.001 |
| **_hatsq** | 0.127 | -0.051-0.077 | 0.697 |
| _cons | 0.003 | -0.151-0.157 | 0.970 |

Note: model is correctly specified if **_hat** is statistically significant and **_hatsq** is statistically not significant (source: <https://stats.idre.ucla.edu/stata/webbooks/logistic/chapter3/lesson-3-logistic-regression-diagnostics> ).
